# Keep Your Options Open: An Information-Based Driving Principle for Sensorimotor Systems

**DOI:** 10.1371/journal.pone.0004018

**Published:** 2008-12-24

**Authors:** Alexander S. Klyubin, Daniel Polani, Chrystopher L. Nehaniv

**Affiliations:** 1 Adaptive Systems Research Group, School of Computer Science, University of Hertfordshire, Hatfield, Hertfordshire, United Kingdom; 2 Algorithms Research Group, School of Computer Science, University of Hertfordshire, Hatfield, Hertfordshire, United Kingdom; Indiana University, United States of America

## Abstract

The central resource processed by the sensorimotor system of an organism is information. We propose an information-based quantity that allows one to characterize the efficiency of the perception-action loop of an abstract organism model. It measures the potential of the organism to imprint information on the environment via its actuators in a way that can be recaptured by its sensors, essentially quantifying the options available and visible to the organism. Various scenarios suggest that such a quantity could identify the preferred direction of evolution or adaptation of the sensorimotor loop of organisms.

## Introduction

In view of the richness and complexity of behaviour of living organisms, one is interested in formulating basic principles that guide adaptive behaviour in organisms. Virtually any decision by a living organism involves the processing of information. Thus, information (in the quantitative sense of Shannon, which we assume throughout the paper) is increasingly being identified as a key property and resource in biological organisms.

A basic formulation of this principle is the *Law of Requisite Variety* [1], [2]. Links between neural complexity and information have been identified [3], [4], and recent work aims at modelling the information processing throughout the perception-action loop of agents [5]–[11].

The hypothesis that quantitative informational principles play an important role is supported by mounting quantitative evidence that organisms are investing a considerable amount of metabolic energy to acquire or process information [12]–[18] A further link in a more concrete setting is provided by recent models which indicate the existence of Bayesian inference mechanisms in the brain which are, in turn, directly driven by a free energy principle [19].

Information is relevant for finding food, navigation, and learning from experience and different types of communication [20], [21]. It has been hypothesized that organisms would derive a selective advantage by optimizing the organization of their sensory and neural information processing according to suitable informational criteria [22]–[25]. The criterion of information optimization provides a biologically plausible structuring power [26]–[28] and could provide significant constraints on the possible structure and dynamics of sensorimotor loops of viable organisms arising from the process of evolution and development.

A particular interest lies in studying how structured behaviours in organisms can emerge under the comparatively sparse feedback provided by evolutionary selection. Typical for the modelling of appropriate self-motivated learning and adaptation mechanisms is the absence of an explicit external goal.

Homeostasis and its generalizations have been proposed to model such mechanisms [29], [30], including information-theoretical criteria such as predictive information [31], [32]. In addition, relevant criteria have been generally motivated by the “flow” idea to find a suitable balance between surprise, challenge and predictability, from the fields of psychology [33], machine learning [34]–[36] and related fields [37], [38]. A central observation is the importance of embodiment for the emergence of intelligent behaviour [39].

In the present paper, we investigate an information-theoretic quantity, *empowerment*, as a hypothetical candidate for a possible optimality principle behind the evolution and development of sensorimotor loops. We explore the plausibility of this hypothesis in a set of different scenarios, involving the discovery of “interesting” world states, simple homeostatic control, the evolution of a sensor, and the emergence of context concepts in a Sony AIBO robot, all arising from the same principle.

## Methods

We study *empowerment*, an information-theoretic utility function which is universal in the sense that it is independent from a specific external task and derives solely from the properties of the perception-action loop of an organism or agent and its interaction with the environment. Empowerment was introduced in its context-free form in [40]. It measures the capacity of the agent to influence the world in a way that this influence is perceivable via the agent's sensors. Concretely, we define empowerment as the maximum amount of information that an agent could send from its actuators to its sensors via the environment, reducing in the simplest case to the external information channel capacity of the channel from the actuators to the sensors of the agent. For a more general consideration, empowerment needs to be formulated using the perception-action loop and information flow formalism from [5], [9], [41] based on the framework of Causal Bayesian Networks [42]. We are looking for a principle that would, in absence of overwhelming imprinted drives, provide natural behaviour preferences that might contribute to modulate pre-imprinted drives or help constituting generic homeostatic variables.

The study of such quantities is motivated by the ability of organisms to select, from a large variety of possible behaviours those that help them survive and adapt in a hostile world where a single wrong action can lead to death. While one can expect evolution to create biases towards certain types of behaviours, still the populations available to the search process of biological evolution are finite and often remarkably small compared to the space of possible behaviours. An individual agent or an agent population can attempt and explore only a small fraction of possible behaviours during its lifetime.

However, the environment in which organisms have to prevail is not random, but intricately structured. Different kinds of models have been devised to capture this structure (e.g. [43]). Nevertheless, the existing domains differ significantly depending on complexity, niche and embodiment of the organisms considered. The present paper suggests that it could be possible to formulate utility functions which are both *universal* and *local*: universal in the sense that they are determined for different scenarios and agents in a unified way and that they are relevant to a wide range of scenarios, species, and ecological niches; local in the sense that they are derived for agent states only inside a limited time horizon from the current one, and thus able to provide swift feedback about the utility of the present state.

If candidates for such *universal utilities* could be shown to exist, they could provide organisms with a “guide” for survival-relevant behaviour wherever more specific drives have not (yet) been established. During evolution such quantities may then “crystallize” into specialized established drives if they turn out to be relevant. This then may provide a principle that helps to discover relevant drives in evolution and development.

If one considers specific utility measures in various (including biological) scenarios such as the nutrient concentration around a bacterium, the social status of a chimpanzee in a group, or the money in a bank account of a person, they have in common that they quantify the options available to an organism or agent. In Gibson's ecological approach where an agent views its environment through perceptions and actions only [44], this can be interpreted as the actions that an agent can perform and whose outcome it can perceive through its sensors.

Under this perspective, we suggest using a generalized measure of *mobility* as universal utility; mobility is known from game-theoretical scenarios as the number of distinct actions that an agent can select in a given situation. It counts the options of an agent. Mobility is known as a powerful heuristic for a number of strategy games (e.g. in the board game of Othello [45]). Also the previously given examples of specific utility functions can be interpreted in terms of mobility. To a sugar-feeding bacterium, high sugar concentration means longer survival time and hence more possibilities to move to promising locations and a higher chance for reproduction, to a chimpanzee higher social status means more mating choices and interaction, to a person more money means more opportunities and more options.

All above examples comprise a drive towards states providing more options, i.e. with more potential for control or influence. To capture this notion quantitatively as a proper utility function, we quantify the control an organism or agent has over its environment. In the spirit of Gibson's ecological approach, this control is measured with respect to what the agent can actually observe.

To cast this into a precise quantitative framework, we use the language of information theory. We measure how much an agent can do and *perceive to be doing* by measuring how much information the agent can inject into the environment and recapture via its own sensors. Importantly, the information an agent injects into the environment is viewed solely in the light of what it can perceive itself, and we distinguish this from what other agents or an all-knowing observer would detect. The concept of “the environment” becomes thus a by-product of the interplay between the agent's sensors, actuators, and morphology. The utility function we envisage becomes an intrinsic property of the individual agent's perception-action loop [46]. We suggest that this quantity, *empowerment*, is a natural candidate for a universal utility and investigate its properties in a representative selection of scenarios.

### Context-Free Empowerment

Empowerment quantifies the agent's *potential* ability to influence the environment as measured by the capacity to “imprint” information onto the environment and later perceive the information via the sensors. To measure this potential it is necessary to disregard the actual behaviour of the agent and to model how the agent could behave in principle (disregarding the actual behaviour of the agent can be imagined as removing the agent's controller and studying the remaining “empty shell” which is the agent's body).

To do this, we formulate empowerment in an interventional framework based on causal Bayesian networks [42], [9]. Here, we require the tracking of *maximum* potential information flow through the system. This is complicated by the fact that Shannon information is not additive. Therefore, for the purposes of quantifying empowerment we provide the agent with an unlimited source of unique randomness, i.e. which is uncorrelated with anything else in the system. This allows us to track its flow through the system since, wherever correlation (i.e., mutual information) is found with the uncorrelated source, it must have flowed there from that source (an approach to measure actual - as opposed to potential - information flow is given in [41]).

The model is based on the causal Bayesian network model of the perception-action loop of an agent in an environment [5], [9]. To define the concept of empowerment in terms of a maximum potential information flow, we first consider the simplest case of the perception-action loop of an agent where the action selection has been disconnected from the sensor input and is made independently from it, specifically through the source of unique randomness. We measure then how much information can at most be sent through the environment from the actuators.

**Figure 1:**
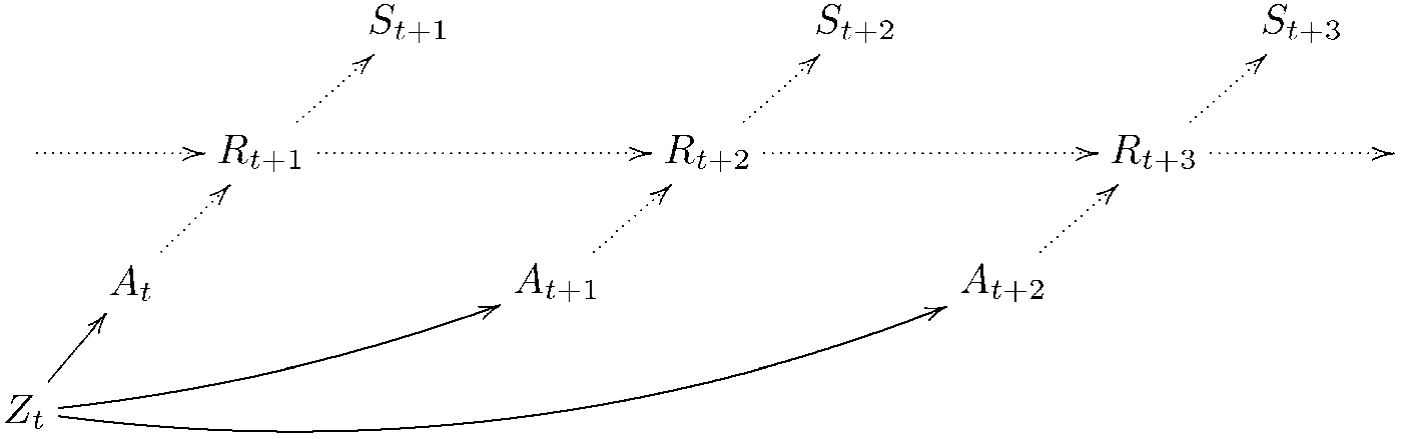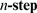 empowerment as the maximum information flow from the actions 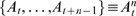 to a sensory input variable 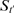 at time step 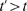. The maximization occurs over the joint distribution of the actions (indicated by the common parent 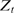). The sensor input that the agent itself perceives later is shown in the top row of the diagram as it is detached from the action selection used for empowerment.

Figure 1 shows the corresponding Bayesian network: 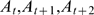 are random variables denoting the agent's action at time 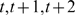, analogously 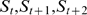 denote the agent's sensor states and 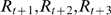 the state of the environment at the corresponding times, and 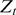 the source of independent randomness, by which actions are selected. In the Bayesian network formalism, each arrow is to be interpreted such that the variable depends only on the probability conditional conditioning of the target random variable on the variables at the origin of its incoming arrows; for instance 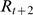 depends on the other preceding variables only according 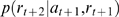; in our model, we interpret the arrows more strongly as actual causal mechanisms, not just as observed probability conditionals [42].

To measure empowerment, we are interested in maximizing the information flow that the actuators can transmit to the sensors at a later time step. For instance, if we consider 3-step empowerment in Fig. 1, we are interested in asking how much information could be potentially generated via actuations 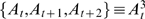 and sent to the sensor 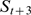 via the environment. This is measured by maximizing the information flow over all independent “free will” distributions 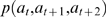 over the actions. To make clear in the Bayesian network formalism that we perform the maximization over a *joint* distribution of actions, we introduce 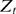 as a joint ancestor over the actions in question.

We thus obtain *n*-step empowerment as
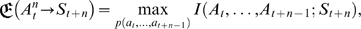
(1)under the Bayesian network from Fig. 1. For random variables of finite size (which we assume throughout), this quantity can be computed by standard channel capacity optimization algorithms. For this purpose, the causal effect of the actions on the sensor values has to be measured.

The causal effect can be measured in general by using independent random actions to probe independent samples of the channel or by computing the full Bayesian model for the channel. However, under certain conditions it can even be attained without probing or intervention, using only observational data with the help of Pearl's interventional calculus [42]. A useful special case of this is given in Appendix S4; it includes scenarios where the whole system state at successive time steps can be observed, such as is the case e.g. in simulations. Once the causal effect is known, the channel capacity is computed directly using the algorithm of Blahut [51].

Note that the quantity from (1) is *open-loop* as it does not utilize feedback from sensor inputs after *t* to select its actions. Rather, the actuation distribution is selected “blindly”. It is possible to generalize the approach to utilize feedback, though the computation becomes more intricate. Similarly, one could consider *interleaved* variants of empowerment where one considers a sequence of sensor readouts 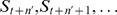 where 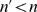, i.e. the readouts begin before the actuations are over. This will, however, not play a role in the following discussion.

Note that the time horizon *n* captures the foresight of the empowerment measure under the influence of the agent's actions. Only features of the environment that can be reached by the actions inside this time horizon are detected by the measure. If the environment is relatively homogeneous and limited, empowerment grows only slowly with the horizon; for instance, in a fully observable deterministic finite-dimensional grid world, empowerment grows only logarithmically with *n*, but any inhomogeneity (such as a locally increased set of options, e.g. induced by the presence of an object that can be manipulated by the agent) reached by the growing horizon is detected by a jump in the empowerment value [40].

### Contextual Empowerment

The previous section assumes that the environmental states from which the agent starts when measuring the empowerment are distributed according to some given probability. It does not distinguish these states in the empowerment calculation. As opposed to that, consider now for a moment the agent's empowerment when starting in different specific states. One finds that, in general, empowerment as well as the action distribution for which it is achieved varies from state to state. In this case the action distribution which maximizes information flow can vary for each individual state, and thus this state-specific empowerment is never smaller (but in general larger) than the empowerment value from last section which was calculated for a global distribution over the states.

Formulating this state-specific empowerment implies access to “objective” state information. As opposed to that, one could consider the empowerment value attainable under the weaker condition of “subjective” information which is limited to the agent's sensoric history or part thereof. The advantage of the latter is that it would be, at least in principle, available to the agent itself. Both “objective” as well as “subjective” variants of empowerment will be considered in the paper; if the agent sensor has access to full state information, they coincide. And both are important special cases of the more general concept of contextual empowerment which we proceed to define in the following.

Define a *context*, denoted with a random variable 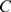, as a collection of random variables from the causal Bayesian network which are non-descendants of the variables in 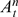 (and 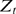). Define the *empowerment given a context* 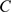, denoted by 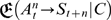, as the average empowerment when context 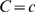 is observed, weighted by the probability of observing a particular realization of the context:
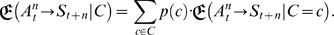
(2)


This can be interpreted as the channel capacity with *side information* known to the sender (actuators) and the receiver (sensors) [47]. Observing the context never decreases the non-context maximum information flow, since the latter can be treated as a special case where the side-information is not used:
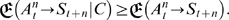
(3)


The world information that an agent itself can access to increase its contextual empowerment is filtered through the sensors and, in general, limited. The most informative context for the agent would be the global state 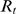; if that is not available, the next best context consists of the sequence of actions and the sensor inputs 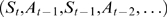 going into the past. This history of the agent's interaction with its environment constitutes an upper limit on what the agent can possibly know about the momentary state of the world.

The state space of such a history, however, can become extremely large. Therefore, for more practically relevant situations we will propose the construction of a *context automaton*, a finite state machine with limited memory, denoted again with a random variable 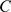, from empirical data. Its purpose is to provide an approximative context for contextual empowerment. At each time step the action 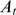 taken and the resulting sensor input 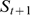 are fed into the automaton. One now searches for an automaton which incrementally and efficiently filters and compresses the history of the agent's interaction with the environment in a way that the state 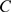 of the automaton, used as a context for empowerment, yields a maximum increase of the contextual empowerment over the context-free case. We will use such a construction as a context automaton. Context automata are a generalization of the concept of 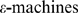 [48], [49], parametrising the state transitions via freely selectable actions.

The efficiency of the context automaton found can be evaluated internally by the agent: it is the difference between the contextual empowerment and the context-free empowerment. Both quantities can be estimated without referring to any variables “outside” of the agent and allow a fully “internalized” formulation of a context automaton which can be found *in an unsupervised way*.

## Results

We now adopt empowerment as a utility defined for each state (context) of an agent and study how it shapes the agent's state space. As a utility, we consider empowerment as guiding the agent to particular preferred states, either by internal behaviours of the agent or by external pressures (say, evolution). Note that if empowerment maximization is to be achieved by the agent's internal behaviours, the agent needs to keep track of its context.

We argue that empowerment provides a natural a priori utility function for an agent by studying a variety of disparate scenarios. While there is nothing special in applying specially designed utility functions to optimize the behaviour of an agent with respect to a task, empowerment is *universal* in the sense that it is always, no matter what the scenario, defined in the same way via the sensorimotor loop of the agent in its environment. In other words, for a given perception-action loop and environment, empowerment systematically induces natural preferred states and, consequently, (as we see in the pole-balancing scenario below) behaviours. Universality is a key property of any viable model of self-motivated or task-independent organism behaviour. In the philosophy of universal utilities, any more specific task-dependence sits on top of these natural tasks or ultimately emerges from them.

In [40], it has been shown that empowerment as a utility is consistent with specialized measures of favourable system states and that it also attracts an agent e.g. to world states where manipulable objects are present in simple grid worlds. Here, we consider several more intricate and relevant scenarios, beginning with pole balancing.

### A Pole-Balancing Scenario

A central claim is that empowerment strives towards states and behaviours one would intuitively classify as “interesting” and “challenging”. To investigate this, we study a simple continuous dynamic task. Pole-balancing on a cart is a classical task from control theory widely used as a simple testbed for various control or learning algorithms. We use this well-known continuous model as it is intuitive and easy to relate to the results. In this section, we compute empowerment for the state space of the pole-cart system and investigate the behaviour that results from local empowerment maximization.

The pole-cart system consists of a wheeled cart that moves along a straight and level track. A pole is hinged to the top of the cart. A force can be applied to the cart, pushing it one way or the other along the track. Conventionally, the goal is explicitly given and consists in applying (or learning to apply) the force as to keep the pole close to vertical. Here, however, we instead use only empowerment as the utility to be maximized by the behaviour of the system. Note that, once the system dynamics, as well as actuators and sensors are given, empowerment is defined in the usual, universal, way. Beyond that, no system-specific goal is given, in particular no indication of the task that one intuitively expects to be solved.

The pole-cart system is described by the cart's position *x*, the cart's speed 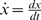, the pole's angle from the upright position 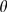 (clockwise), and the angular speed of the pole 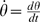. The variables are related as following:
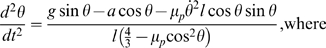
(4)

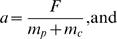
(5)

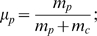
(6)

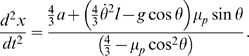
(7)


We use the constants given in Table 1 for our experiments. The Euler integration method with step size 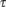 is used for updating the state of the system through time. Although this method is imprecise and unstable [50], we employ the method for consistency because it is commonly used in the majority of treatments of the pole-balancing problem. In any case, we do not expect the essence of the results to significantly depend on the precision of the integration method.

With the Euler integration, the state of the system at simulation time step 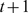 depends on the previous state at time step 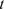 as following:
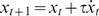
(8)

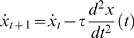
(9)

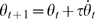
(10)

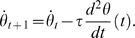
(11)


If 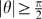, then 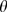 is clipped to either respective boundary 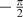 or 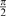, and 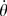 is set to 0 – after falling onto the cart, the pole cannot continue falling further. Note that for simplicity and consistency with the diagrams such as Fig. 1, *t* denotes just the time index step, not an absolute time measured in time intervals *τ*.

Consider an agent which can apply force to the cart, and can observe the results. We now study how the agent's empowerment depends on the state of the pole-cart system.

Available to the agent is the action of applying the force *F* or −*F* to the cart. Each action lasts for the time 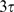 and the force is kept constant during that time. Assume that, starting at time step 0, the agent performs a sequence of 10 actions, denoted by a random variable 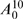. After this sequence of ten actions the agent observes part of the state of the system, namely the angle of the pole 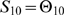. To calculate empowerment, the angle variable 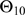 is discretised into 101 equal bins (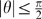 rad). Using this model, we will calculate 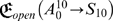 for different initial states of the system.

The system dynamics is sensitive to initial conditions. Therefore, instead of initial states concentrated on one point, we need to consider slightly perturbed ensembles of starting points to obtain representative trajectory distributions. For an initial state 
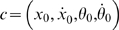
 the ensemble consists of 5^4^ states uniformly distributed around the state in the 4-cube with side 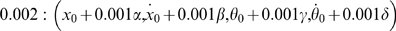, where 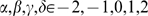. This ensemble is then used compute the causal effect 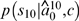 by obtaining 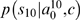 for each of the 2^10^ action sequences. Open-loop empowerment 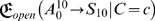 is then computed from the causal effect as the channel capacity of the channel 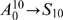 characterized by 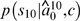. The capacity is found with a precision of 10^−4^ bit using the iterative algorithm by Blahut [51].

**Figure 2:**
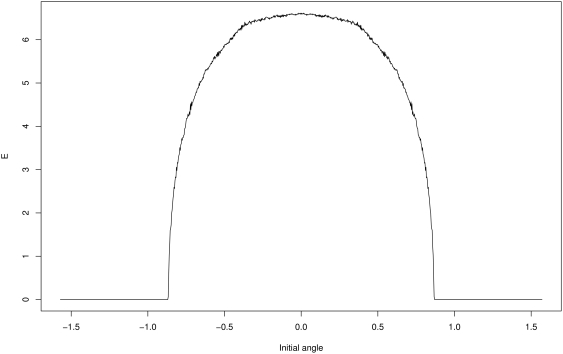
Empowerment 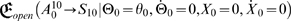 for 1001 equispaced initial angular deviations of the pole from the vertical: 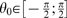 rad.

Figure 2 shows how empowerment depends on the initial angle 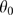 when the other three system variables start at zero. There is an abrupt cutoff angle after which empowerment drops to zero. If the pole starts at more than the critical angle from the vertical, the applied force is not enough to influence the pole's final position after 10 time steps — the pole falls regardless of the agent's actions. The small deviations from monotonic growth of empowerment as one moves towards 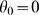 are not just due to sampling error but also likely to partly arise from branching points in the dynamics of the system.

**Figure 3:**
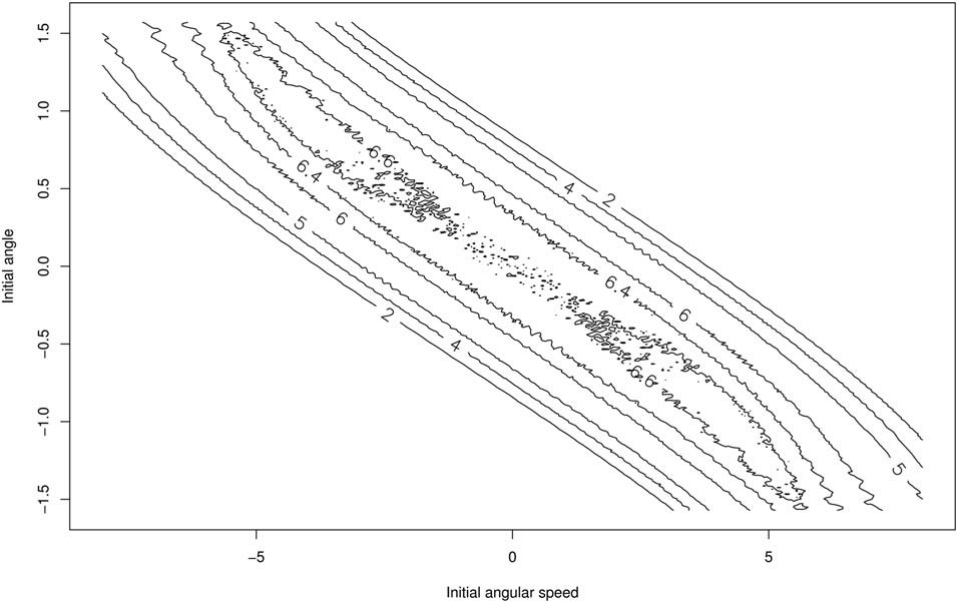
Contour plot of empowerment 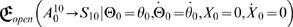 as a function of the initial angle 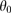 and the initial angular speed 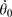 when the initial position and the speed of the cart are 0. Obtained for 301×301 equispaced initial positions 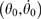.

If only the initial angle is varied while the other variables are fixed to zero, then empowerment is highest for 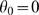, i.e. for a pole that starts vertically. This corresponds to the intuitive and natural upright target state of conventional learning tasks. Interestingly, the full picture becomes more intricate if we allow the initial angular speed also to be varied. In that case, 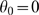 is no longer the state with maximum empowerment and, as Fig. 3 shows, there exist states away from 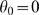 with slightly larger empowerment (by 0.07 bit). These “off-centre” states correspond to a pole which is leaning to one side, but which has an angular speed that would quickly turn it towards the upright position (and ultimately beyond). The results show that this special constellation allows the system to reach a (slightly) larger variety of states in 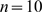 action steps than the upright pole with zero angular speed. This saddle-point property is robust with respect to the variation of parameters of the cart-pole system. However, it depends on including the cart in the model, as the effect disappears in a pure pole-control scenario (without cart and with only torque control at the base of the pole).

Thus, if we would selectively poise the system in these states, it is these off-centre states that would turn out to have the highest empowerment (which is not immediately expected). However, once the system itself is required to attain maximum empowerment states, the situation changes.

In fact, it turns out that above “off-centre” maximum empowerment states are unreachable through the intrinsic dynamics of the agent: while one can externally set up the initial state carefully to start in these states, it is not possible to devise an internal action strategy that is able to reach them under the given actuator dynamics. These states are similar to Garden of Eden states (i.e. states with no predecessor) in dynamical systems in the sense the agent has no way to reach these “off-centre” maximum empowerment states by itself in a sustained run under its own dynamics.

The picture is completed by considering the behaviour of an agent that is entirely guided by its local *n*-step empowerment. Assume that the agent controls the cart-pole system at each time step as to maximize the 10-step empowerment of the following state. As before, assume that the only two actions available are applying the force of either −10 *N* or 10 *N* to the cart.

**Figure 4:**
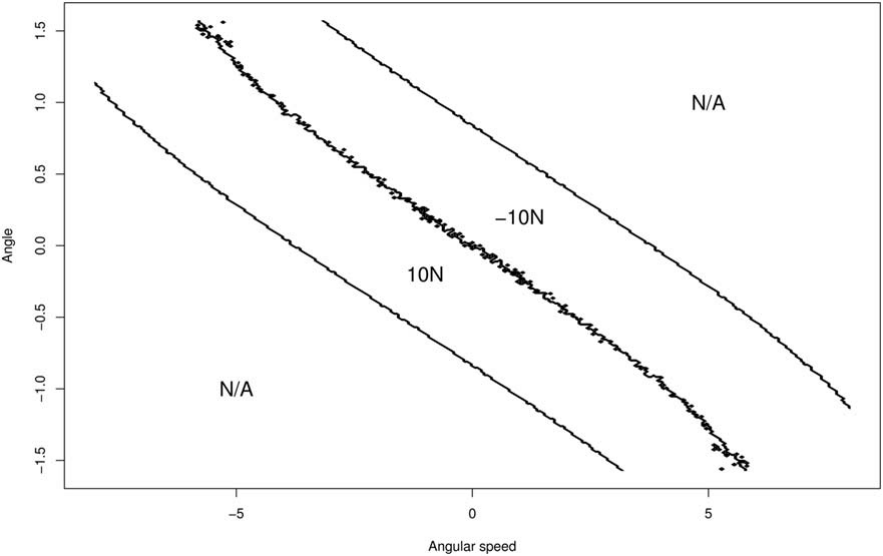
Action selection policy for maintaining high expected empowerment as a function of the angle 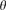 and the angular speed 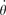 of the pole. Obtained for 301×301 equispaced states. In the areas marked with “
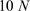
” and “
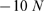
” the corresponding action should be chosen, whereas in the two areas marked with neither of the actions can prevent the pole from eventually falling.

We base the control policy on just the pole's momentary angle and angular speed since the control model can be simplified by transforming the system into one where the cart's position and speed are zero. We now consider the control that greedily maximizes empowerment, as follows: for each combination of the pole's angle and angular speed we determine the successor state that results when each of the available actions −10 *N* or 10 *N* are applied. For each of the possible successor states we determine the 10-step empowerment. The greedy controller then selects that action that results in the successor state with the highest expected empowerment. Figure 4 shows the resulting action selection policy.

When the system is run under this greedy policy of empowerment maximization, it turns out that the system indeed performs classic pole-balancing (note that this works also on a moving cart since the dynamics of the system is invariant to constant velocity shifts). There is an expected and a less expected aspect to this observation: on the one hand, a priori pole-balancing appears to be an intuitive and natural task in the cart-pole system. It coincides with a high level of options available to the agent by the pole being poised in the unstable upright position.

However, note that, on the other hand, empowerment is, while quite close to the maximum, not strictly maximal at the upright position 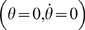, as we have seen in Fig. 3, since there exist higher empowered states. These are however excluded by the combination of the condition of a continuous running system constraining the states which can be reached and reliably maintained over time with the greedy policy for empowerment maximization. Together, they manage to ultimately reduce the dynamics to the fundamental pole-balancing problem. The “off-centre” states that would theoretically maximize empowerment cannot be realized nor sustained.

Furthermore, it should be noted that this greedy policy is local in the following sense: there is a one step look-ahead on which action selection will maximize 10-step empowerment, and the 10-step empowerment has a look-ahead of 10 time steps, so the policy has a local time horizon of 1+10 steps. However, there is no “overarching plan” to balance the pole. This global behaviour emerges purely from the local empowerment-maximizing dynamics of the system (this can be compared to the globalistic structure of reinforcement learning models where not only the agent rewards have to be explicitly designed, but where they also essentially have to be propagated throughout the system to all states [52]).

Again, it should be noted that there was no scenario-specific hand-crafted task to balance the pole. The complete behaviour derives from the generically formulated empowerment-maximizing policy, defined exactly same way as it would be for any other system under consideration. We will highlight possible implications of this property for homeostatic dynamics in the Discussion.

### Empowerment-Driven Evolution of Sensorimotor Apparatus

While artificial systems typically have fixed sensors and actuators, in biology these evolve over time and cannot be assumed to stay unchanged. The evolution of sensors and the sensorimotor loop has been hypothesized to be an important driver of evolution [53]. An organism's sensorimotor loop contributes significantly to its success.

The question emerges whether it would be possible to quantify the advantageous contribution of a particular sensorimotor loop more immediately than can be achieved by the delayed evolutionary feedback via selection. The importance of information discussed earlier in the Introduction indicates that it could be expected to be suitably correlated with an overarching fitness advantage [54], [55], [56], [18].

We now use empowerment to immediately measure the quality of a perception-action loop and feed it directly as a fitness criterion into a simple artificial evolution model. Any further constraints on the agents are summarily captured by constraining the possible structure and informational bandwidth of sensors and actuators. This experiment extends earlier results from [56] and studies the nature of the qualitative change of the resulting sensoric morphologies as experimental conditions are gradually modified.

To apply empowerment in this scenario, one should note that it strongly depends on the causal effect of the agent's actions on the future sensor input. The causal effect (for a brief definition of causal effect, see Appendix S3 or, for a detailed exposition, see [42]) is a function of the sensory mechanism 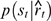 and the actuatoric mechanism 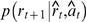 which can be interpreted as a description of the agent's embodiment: a description of how the agent's sensors and actuators interact with the environment. Thus a way of modifying empowerment is to modify these mechanisms. Depending on the concrete scenario, this can be realized as a modification of the environment, the agent's morphology and actuators, the agent's sensors, or all of them.

This section illustrates the idea specifically by evolving sensors and actuators of an agent for different locations (i.e. contexts) in a simulated world. Note that empowerment can be considered to provide local and immediate feedback to the evolutionary dynamics — our present evaluation is based on the short-term value of empowerment rather than some longer-term delayed fitness measure such as the survival of the agent in the environment.

Consider an infinite two-dimensional square grid world. A source is located at the centre of the grid. The source emits a signal, the strength *P* of which in any cell of the grid is 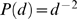, 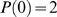, where *d* is the Cartesian distance from the source.

An agent moves in the world occupying one cell at a time. The sensor model consists of a number of detectors at fixed positions relative to the agent. Each of these detectors samples the strength of the signal *P* their position around the agent. The sensor then identifies the one with highest strength. If several detectors measure a maximal signal, the tie-breaker is to pick one at random, with uniform probability. The available repertoire of detectors around the agent will be varied according to the scenario below.

Similarly to the detectors, the agent has a given repertoire of actions. An action is a jump of the agent into a cell relative to the agent (not necessarily just neighbouring cells). In addition, the agent can choose as action not to move at all. We shall now present two complementary scenarios: evolving a sensor for a given actuator, and evolving an actuator for a given sensor.

Assume that the agent's actuator has a fixed repertoire of just five actions: stay in the current cell or move into one of the four adjacent cells south, east, north and west (von Neumann neighbourhood). We now investigate the best layout of the sensor detectors so that the agent's *n*-step empowerment is maximal.

For the experiment, we constrain the set of sensors to those finding the cell with highest signal strength near the agent. As mentioned above, each sensor is modelled as a set of sampling points (detectors) arranged relative to the agent. For example, a sensor measuring the local gradient surrounding the agent using the von Neumann neighbourhood consists of four sampling points:{(0, −1), (1, 0), (0, 1), (−1, 0)}, denoting the cells directly south, east, north and west of the agent to be sampled.

To find good sensors we search in the set of sensors using an evolutionary algorithm. The algorithm treats each sensor layout as an individual. The sampling points of any sensor are constrained to lie within a fixed square with side *b* around the agent. The maximum number of sampling points a sensor can have is fixed. At any point in time, the returned state of a sensor identifies that sampling point which measures the highest signal strength. Hence, the number of sampling points is also the number of states of the sensor 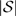.

We define the fitness *F* of a sensor as the 4-step open-loop empowerment 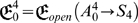 of the agent equipped with the sensor. The fitness also includes a small penalty for the number 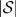 of sampling points used:
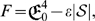
(12)where we set 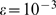. The penalty serves to select, among essentially equivalent sensors those which are more economical and have fewer sampling points. The exact value of the penalty used here is arbitrary, though small.

The fitness of a sensor is evaluated for a particular initial position of the agent in the world (for instance, the centre which is at (0,0) in Cartesian coordinates). The required (interventional) conditional probability distribution 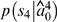 that describes the resulting state 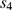 of the sensor at the fourth time step after carrying out the four actions is calculated exactly from the Bayesian network [42]. The 4-step open-loop empowerment is then the capacity of this channel characterized by the interventional probability distribution. The capacity is found with 10^−4^ bit precision using the iterative algorithm by Blahut [51].

We initialize the population with five randomly generated sensors. In every generation, the five best sensors produce five offspring each. The size of the population is between 5 and 30. Five best individuals from the parents and offspring are selected into the next generation.

An offspring is produced from its parent by mutation. The mutation operator supports two operations: (1) addition of a sampling point, and (2) deletion of an existing sampling point. If the sensor has no sampling points, the mutation operator always adds a point. If the sensor has the maximum number of sampling points, the mutation operator always deletes a point. Otherwise, either a new point is added or an existing one is deleted with equal probability.

To speed up the search and make it more efficient we have incorporated ideas from simulated annealing and tabu search: (1) the number of mutations performed is uniformly distributed between 1 and 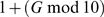,where *G* is the generation number; and (2) we do not add offspring which have been evaluated before or are already present in the population. To sample the solution space thoroughly we run the evolutionary algorithm at least 10 times for 1000 generations each. The best individuals are selected across these runs.

We have evolved the sensor for different positions of the agent in the world to illustrate how empowerment makes sensors and actuators adapt to the niche in which the agent exists. We have constrained the sensor to a maximum of 20 sampling points which lie inside the square with side length 21 centred around the agent.

**Figure 5:**
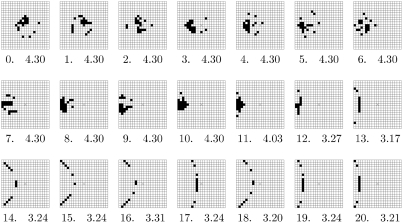
A possible phase transition in the layout of best evolved sensors. Best evolved sensors for position 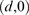 are shown above, where 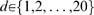. The transition from clustered to arched layout occurs around 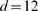.

Figure 5 shows the best evolved sensors for different starting positions of the agent. The positions shown begin with the agent at the centre (denoted by distance 0 to the centre), as well as displaced to the east by 1, 2, …, 20 cells. The artificial evolution found essentially two qualitatively distinct types of sensors. For an agent located near the signal source (up to a distance of 11 cells) the sensors form nearly two-dimensional “blobs” which capture more or less the absolute displacement from the source. However, for the areas further away from the source (12 cells and more), the sensors change character and collapse into roughly one-dimensional circular segments centred approximately at the source and which only capture the bearing to the source. This phenomenon appears consistently also for other displacement directions (not shown here), not only for vertical ones which are equivalent to the horizontal ones due to symmetry, but also diagonal ones.

In earlier experiments investigating agent displacements 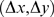 from the centre [57], it turns the best sensor layouts for starting positions (20,0), (10,10), (20,10), and (20,20) are stable in the sense that, if the evolutionary experiment is repeated, the resulting solutions remain almost exactly the same. Common to these positions was that they are relatively far away from the centre. Evolved sensor layouts for agents closer to the centre, e.g. (0,0) and (10,0), are much more prone to variations while retaining exactly the same empowerment and hence fitness. This suggests that these solutions belonged to a plateau of the fitness landscape that allows for some significant variety of optimal solutions. Qualitatively, though, these layouts still consisted approximately of a blob of sampling points. The blob was typically centred at the agent for the (0,0) starting position, and on the far left for the (10,0), essentially covering the centre of the gradient field.

We now inspected the population of the evolutionary algorithm more closely. This revealed that even in a distance *d* up to 11 from the centre individuals with arced sensors can already be found in the evolved population. However, with their smaller empowerment value, they are inferior to the blob sensors which constitute the best solutions in this case. As *d* grows beyond 11, the advantage of the blob sensors vanishes and the arced solutions start dominating. This indicates a replacement of one major solution niche by another on approximately continuous change of the parameter *d* and this, in turn, a phenomenon analogous to a phase transition in the transformation of the blob into the arc.

Since in the discussion we will return to this sensor evolution scenario in a broader context, we briefly conclude the section by mentioning the results from [57] concerning the complementary task of evolving actuators which maximize empowerment for a fixed sensor. Apart from that, that experiment was similar to the sensor evolution experiment described above. The main observation was that, unlike the sensors, the evolved optimal actuators exhibited a significant variety.

Summarizing, this section demonstrates that empowerment can serve as an immediate guide for sensor and actuator evolution. In particular, using empowerment as a fitness function allows evolution to implicitly switch from one qualitative representation of information to another one, namely from a “blob” sensor measuring an absolute displacement vs. a bearing sensor in the sensor evolution experiment. The switching was emphatically not designed into the model but emerged through the empowerment optimization and via a discontinuous shift from one solution niche to another in the manner of a phase transition. In the Discussion, we will present possible insights this may provide into the abundance of sensory modalities in nature.

### Relevant Contexts Induced by Empowerment

When considering contextual empowerment, we noted that, if a context helps increase empowerment, then we expect the context to be sharing information with the global state of the system. This makes it possible for an agent to obtain an intrinsic meta-sensor for features in the global state of the system that are relevant to empowerment, purely by constructing a suitable context which increases empowerment.

In this section we illustrate this idea using a hardware robot, the Sony AIBO. The intention is to demonstrate that empowerment, while being a completely intrinsic function, can be used to *construct* contexts which correspond to external concepts, and can thus assign an analogy of “meaning” [58]–[60] to the robot's actions.

**Figure 6:**
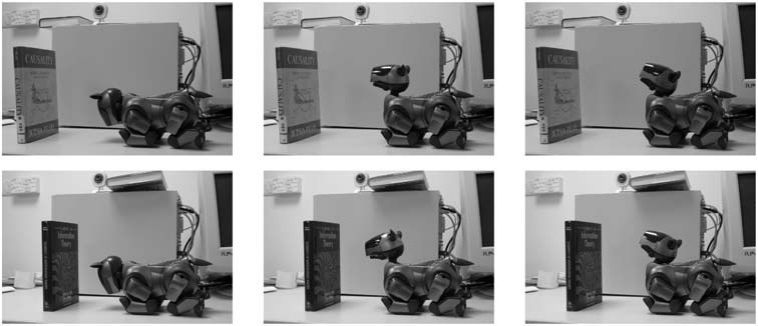
Setup of the experiment with the AIBO. From left to right the photographs show the minimal, zero, and maximal tilt of the AIBO's head.

We use an AIBO ERS-210A robot dog. The robot is lying on a desk (Fig. 6). We employ only one type of action, namely setting the robot head's tilt to a value in [−1;1], and concentrate exclusively on the infra-red (IR) sensor mounted in the head of the robot and pointing along the longitudinal axis of the head. The sensor measures the distance to an obstacle in front of the head. The effective range is below 1 *m*. Moreover, the sensor is noisy, for example, because of the reflections from the table.

In this experiment the AIBO performs a randomly chosen action (set the head's tilt to a randomly and uniformly chosen value in [−1;1]) every two seconds, and at the end of the two second period, just before performing a new action, the IR sensor reading is captured (the interval of two seconds is long enough for the effect of an action to be independent of any previous actions. This is to simplify the experiment for proof-of-concept purposes). Every 200 actions a book is placed in front of the robot for 100 actions and then removed. The experiment lasts for 1000 actions.

Based on the captured data we calculate the average empowerment by choosing a quantization of the actions and the sensor inputs (see below for details). We base the empowerment function on the effect of an action on the IR sensor 2 seconds later. Empowerment is increased by the knowledge of whether the book is present in front of the robot or not, because the causal effect of an action on the sensor depends on the presence of the book in front of the robot. The increase is different for different combinations of the quantizations of action and sensory input.

In this experiment we distinguish three empowerment quantities: *context-free empowerment* – empowerment when no context is used, *book-contextual empowerment* – empowerment where the state of the book (the book is either present or absent) is used as the context, and *controller-contextual empowerment* – empowerment where the state of a suitably constructed context automaton (see the section introducing contextual empowerment) is used as the context.

There exists a context (namely, the presence or absence of the book) that increases contextual empowerment. However, the question is whether it is actually possible to create a context automaton which has controller-contextual empowerment that is higher than the context-free empowerment, and whether the state of such a context automaton would actually capture information about the state of the book, thus serving as an indirect sensor for the presence or absence of the book. Since we designed the experiment so that the only external factor that would influence empowerment is the state of the book, we expect this to be the case.

We address these questions by performing an evolutionary search to find a two-state context automaton that provides the highest contextual empowerment and compare the controller-contextual empowerment of the best automata with the book-contextual empowerment.

The action (a tilt in the interval [−1;1]) and the sensor input (a distance in the interval [0;1], measured by the infra-red sensor) two seconds after the action has been taken are quantized into bins of equal size. The number of bins used for the action quantization may be different from the number of bins used for the sensor input. This quantization creates two discrete random variables: the action *A* and the sensor input *S*. The presence or absence of the book in front of the robot is denoted by a random binary variable *B*. The experiment generates a single time series 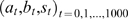 from which all quantities are later calculated.

The underlying causal Bayesian network is 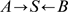. The *context-free empowerment* 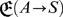 and the *book-contextual empowerment* 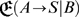 are calculated from the network using the total joint distribution 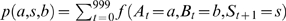, with *f* denoting the empirical frequencies of the given event combinations 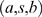.

The context automaton is a deterministic finite-state automaton with a binary state *C* and uses the momentary sensor input and action as input. At each time step *t* the automaton performs a time-independent mapping 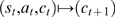. The underlying causal Bayesian network is similar to the above book-contextual case except that *C* is used instead of 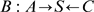. Analogously, we calculate the *controller-contextual empowerment* as 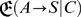.

Due to undersampling, the theoretic assumption that *C* and *A* are independent does not hold in general if the context automaton *C* is extracted from an empirically sampled finite time series. Therefore, when evolving context automata (mappings) to maximize the controller-contextual empowerment, we add a bottleneck-type penalty term [61] to the empowerment value, and use the modified expression 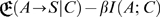, here with β = 1, as fitness function. This quantity penalizes the empowerment for a violation of the assumption of independence between *A* and *C*. The above fitness function is again calculated from 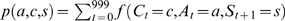, with *f* the empirical frequencies.

**Figure 7:**
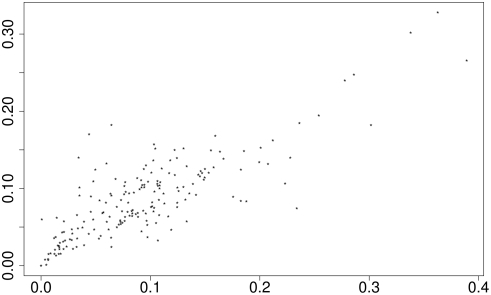
Empowerment gain (in bits) by a best evolved context-automaton over the context-free empowerment (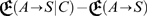 on the vertical axis) plotted vs. the difference between book-contextual empowerment and context-free empowerment (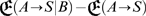 on the horizontal axis).

We performed an evolutionary search for each combination of 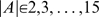 and 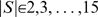 to find a two-state context automaton with the highest controller-contextual empowerment. The context-free empowerment for these cases lies in the interval [0.52;2.03], the book-contextual empowerment in [0.61;2.15], and the controller-contextual empowerment of the best evolved controllers in [0.56;2.15]. As a general trend, it turns out that, whenever there is a significant difference between context-free and book-contextual empowerment, indeed context automata tend to be found which achieve a controller-contextual empowerment close to the book-contextual empowerment (Fig. 7). Thus, the difference between book-contextual empowerment and the context-free empowerment can be interpreted as a potential for evolution to find a context automaton with high controller-contextual empowerment.

**Figure 8:**
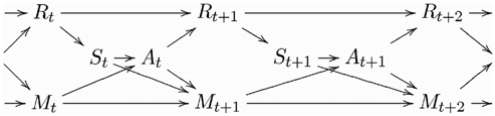
Perception-action loop as a Bayesian network. 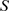
 – state of the sensor, 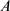 – action performed by the actuator, 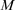 – state of the memory of the controller, 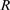 – state of the rest of the agent-environment system. The diagram can be read as follows: action 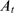 is picked given sensor state 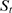 and memory state 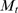, sensor state 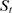 is obtained from the state of the rest of the agent-environment system 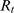, and 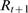 is obtained from 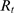 and 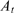.

**Figure 9:**
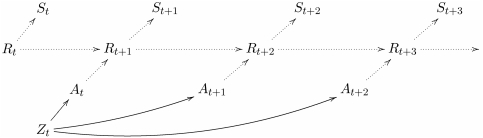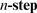 empowerment as the maximum information flow from the actions 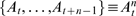 to a sensory input variable 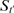 at time step 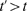. The maximization occurs over the joint distribution of the actions (indicated by the common parent 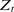). The sensor input that the agent itself perceives later is shown in the top row of the diagram as it is detached from the action selection used for empowerment.

Note that empowerment maximization using the context automaton evolution leads to contexts corresponding to external states, even while it only uses intrinsic data available to the robot. As expected, this only happens when the effect of the robot's actions on its sensors indeed depends on these external states. The evolved context automaton is thus able to reconstruct aspects of these external states. This can be considered as assigning a rudimentary kind of “meaning” to the agent's action-selection or information processing mechanism.

## Discussion

We wish to emphasize several salient points of the empowerment model. First of all, using empowerment to direct task-independent behaviour is distinct in a number of relevant aspects from the other methods suggested for that purpose. For instance, the autotelic principle [38] or the learning progress [37] require measures which are to some degree tailored to the problem at hand and intertwined with the particular learning model. Other perspectives for self-motivated behaviour range from reinforcement learning, to homeostasis and autopoiesis principles [35], [29], [62].

The latter are conceptually closer, as is the homeokinetic approach [63], [64] and a closely related information-theoretic variant of that method, based on predictive information [32] which considers maximizing predictive information in the system, i.e. the information that the sensory past (or a part thereof) of the agent has about its future. Similarly, other information-theoretic quantities operating on the system dynamics, and other coordination measures, as well as excess entropy have been used successfully to study the generation of intrinsically motivated behaviour in embodied systems [65], [11].

As opposed to these measures, empowerment quantifies (1) a *potential* rather than an *actual* information flow in the system, probing the “potentiality space” rather than the actual trajectory as most task-independent functions do; (2), it looks at information that could be generated specifically by the agent itself and then injected into the system, rather than information (or entropy, depending on the perspective from which it is considered) that is generically produced somewhere in the system.

The fact that empowerment deals with what an agent *could do* rather than what it actually *does* is conceptually a significant difference to the other models since it implies that empowerment does not depend on a particular action-selection mechanism or controller. It derives only from the embodiment, i.e. on how the agent is linked into the environment. The other approaches consider the whole system dynamics and link the concrete agent behaviour directly to their utility measure.

Importantly, in empowerment, the actuatoric dynamics plays a role that is explicitly conjugate to the sensory dynamics, whereas most approaches focus on the sensory time series while their actuatorics is essentially implicit. This is due to the fact that, to compute empowerment, the perception-action loop is treated in a fundamentally causal way, in contrast to the other approaches which limit themselves to observational, correlative measures. The advantage is that, by this separation of sensors and actuators, empowerment provides a very transparent measure of the role that is played specifically by the agent as opposed to its environment.

However, this conceptual clarity comes at a price. The downside of using empowerment is that, as a causal quantity, it is significantly more difficult to compute than the other quantities of this kind. In most of the above experiments, empowerment had to be computed using a “detachable” world model that allowed to reposition and retry certain behaviours in a particular situation. An exception to that was the hardware AIBO scenario for the discovery of relevant contexts, where however we had to set up the experiment in a particular way (e.g. waiting for two seconds to make sure that the effect of an action has taken place) and combining it with the context automaton evolution to be able to isolate the causal effect of the actions.

This means that, unlike the other approaches mentioned many of which operate on-line, it is not straightforward to use empowerment to generate per se exploratory or self-adaptive behaviour. Empowerment identifies only the “importance” of system states and does not prescribe *how* to reach them. Obviously, such a rule can be easily derived, e.g. by greedy action selection, as in the cart-pole system. However, to actually compute this, this requires a more or less sophisticated world model. Since the present paper studies empowerment from a proof-of-principle perspective, it is not currently preoccupied with a efficient ways of implementing the model (which are explored in separate work), but rather with identifying the general and fundamental principles at work and whether it makes sense to consider empowerment as a universal utility.

In a variety of scenarios highlighting different aspects of possible agent worlds, we have seen that empowerment identifies intuitively “interesting” aspects. Previous work has shown that empowerment identifies states which increase options e.g. by allowing the manipulation of a box or finding areas in mazes or worlds which strongly correlate with hand-selected performance measures [40], [66].

In the sensor evolution model, empowerment provided a model for the emergence of qualitatively different sensors (blob vs. arc), depending on the state (“niche”) of the agent. Especially important was the cart-pole example in which empowerment provides the system with a natural “pole-upright” homeostatic dynamics without having to specify this explicitly as a task. In particular, while a greedy empowerment maximization was determined locally for each state, it nevertheless resulted in a global homeostatic behaviour. There are some indications that this property generalizes to other scenarios. In the AIBO experiment, we showed that empowerment induces a relevant context from which an agent may be able to induce a subjective “meaning” for certain aspects of the environment.

Importantly, in all these experiments empowerment was virtually defined in the same way. Once the system, the agent, its sensors and actuators as well as their resolution is fixed, then, for a given time horizon, empowerment is determined in the precisely same fashion.

It is not obvious that the states favoured by empowerment should match the intuitive expectation of good states: the consistent observation of this phenomenon requires discussion. Of course, it is easy to design explicit counterexamples: for instance, in the cart-pole system one could imagine the specific task of having the pole drop to the right side. While this is generally easy to achieve, and is an unchallenging task compared to the upright pole balancing, it might be a valid system goal. Note, however, that if the agent starts in a highly empowered state (upright pole), one has no difficulties to bring the system to the desired target of having the pole fall to the right, while the converse is not true.

This principle of identifying generically advantageous initial states is a general property of empowerment. So, while empowerment does not need to be correlated with an explicitly given goal, being in a maximally empowered state is a good a priori guess for an initial state that maximizes the agents' chances of homing into a suddenly emerging goal. It identifies states which allow the agent to “keep its options open”.

This makes empowerment a particularly suitable candidate measure to identify advantageous states in scenarios where the agent has to sustain itself over time — it identifies states of particular sustainability for a given environment and sensorimotor equipment (this is analogous to the role of the free energy principle for the brain from [19] which has been proposed, among other, to prevent phase transitions deleterious to the system's organization). Such a setting, in turn, is highly relevant for biological systems and could lead to understand how homeostatic variables can emerge intrinsically in a biological system with a given sensorimotor equipment. If biological adaptation would indeed aim at maximizing sustainability over various time scales according to a principle such as empowerment maximization, then it might be possible that novel homeostatic variables could emerge from an evolution of regulative processes that implement dynamics similar to empowerment maximization. Thus, this could provide an insight how the evolution of the rich set of homeostatic and regulatory variables on all levels of living organisms is directed, providing a powerful principle for biological self-organization.

It is unlikely to expect that a quantity such as empowerment is measured and optimized directly in biology, i.e. that a strong principle of empowerment maximization would hold. A weak form of the principle, however, might be possible: analogously to the optimized information transmission in neurons [67], [17], [16], over time evolution might result in organisms that implement a suitable informationally optimal dynamics, at least to some approximation. In that case, quantities such as empowerment could provide transparent insight into the selection pressures that guide the emergence of successful information processing architectures on all levels of biology.

The main free variable of the empowerment model is the temporal horizon. Its depth determines not only the “foresight” of the empowerment measure, but also to which extent one can model the focusing on a target. As illustration, consider a predator homing in on its prey: the predator's short-term empowerment is reduced due to its energy and time expenditure, however, on successful capture and consumption of the prey, the predator's long-term empowerment is again increased as its life span is prolonged and, hence, the predator's potential to continue carrying out actions in the future. More generally, if one considers only sustainable systems (the ones typical to biology), then with a sufficiently large temporal horizon the homing in on specific targets is covered by the maximum empowerment principle.

As seen in the sensor evolution scenario, empowerment can drive sensor and actuator “morphology” evolution. Since the value of empowerment involves properties of both the actuators and the sensors, it provides an immediate measure for the efficiency of the perception-action loop and a direct gradient for adaptation without requiring the achievement of specific life tasks for the given organism. Of course, in general there will be metabolic, bandwidth or morphological constraints, which can be readily incorporated in the constraints on sensor and actuator evolution. But, given such constraints, a quantity such as empowerment provides an immediate gradient for the adaptation or evolution of sensorimotor loops. The alternative would be selection via survival, where the success of a particular sensorimotor loop would be only determined after prolonged time and thus would entail indirect and often significantly delayed feedback concerning the performance of a given sensorimotor loop.

The simple principle of empowerment maximization together with the size constraint on the sensor placement in the sensor evolution scenario highlights further interesting aspects: the sensor structure changes qualitatively, from a blob to an arc as the agent moves from one “niche” to another. Thus even this simple model provides an indication how the emergence of a rich variety of biological sensors specifically adapted to their particular sensory niche might be guided by some universal, for instance empowerment-like principle.

On the other hand, this scenario has been shown earlier to exhibit much less selection pressure when actuators were evolved [57]. In this case, many quite different, but equally empowered designs were found. This was due to the fact that the actions were selected freely by the agent (apart from bandwidth, there were no restrictions on the actuators) and thus the actuators had more freedom in *producing* their information than the sensors have in *extracting* information from the environment. This can be loosely interpreted in the way that information that can be produced by the actuators of an agent is of a “higher quality” than that which can be extracted by the sensors; the behaviour of an active agent exhibits a signature. This leads, in turn, to a reduced selection pressure during actuator evolution, as the agent can use the significant control over its actions to compensate differences in actuator setups. In the biological reality, however, actuators suffer from very significant energetic and mechanical limitations and costs. We thus expect that once these factors are included, these will end up producing a significant additional selection pressure on the morphology and operation of actuators. On the other hand, if substantial energetic or morphological constraints are absent, we do indeed predict that the evolution of actuators under empowerment will experience significantly less specific selection pressures than the evolution of sensors.

The experiment with the AIBO robot shows that empowerment is able to provide an agent with an intrinsic concept of relevant features (contexts) in the environment. The context found from a time series observed internally by the robot sensors matched well the externally determined presence or absence of a book. For the presence/absence of the book to be relevant to the robot, it has to have an influence on the robot's perception-action loop. In this view, if the book is not perceived, but also if it does not modulate the influence of the agent on the world, the context change is not considered relevant to the agent.

Returning to the original question, we asked whether it is possible to formulate universal utilities for (specifically biological) agents. If it were possible, this could provide some both universal and local guiding principle for the adaptation of biological systems. We have seen above that there are a number of candidates for this. The present paper, in particular, studies the suitability of empowerment for that purpose.

Such a hypothesis requires a discussion why an evolutionary process should at all, on the long run, end up with some universal utility emerging to guide its direction. While the present paper does not attempt to suggest a particular mechanism by which this could be attained, in the case of empowerment we can propose some hypothetical paths for the emergence of such a phenomenon. Probably the most direct one can be discussed in the context of the homeostatic effect mentioned earlier this section. A sustainable system (as typical for biology) needs to be “prepared” for a certain set of perturbations and needs to be able to counteract them with a maximal probability of success. A measure such as empowerment could then emerge as a result of this selection pressure. Note that this implies that sensors that better identify the dangerous perturbations, and actuators that better counteract them will then also be favoured by the selection process. In other words, the a priori structure of sensors and actuators could incorporate much of the evolutionary “experience” of relevant perturbations, and an agent's life-time adaptation would then just fine-tune the balance by maximizing e.g. empowerment or a related quantity.

Once equipped with certain sensorimotor properties, actions which cannot be distinguished via the sensors, or sensory modalities that cannot be affected by the actions at some time scale are likely to degenerate away, as information processing is energetically costly [12]. It thus makes sense to hypothesize that, in an approximately stable equilibrium of evolution and individual adaptation, an agent will be to some degree in a state of optimality with respect to empowerment; the plasticity of the control mechanism would lead the agent into a state where the actuators can exploit the perceivable sensory bandwidth to the fullest. Where not, one would expect the sensors to degenerate over evolution if they do not provide a selective advantage or the actuators to be enhanced if they do. One mechanism for this could be the discovery of novel modes of actuation and manipulation which provide the agent with additional degrees of freedom.

It should be noted that we say nothing about how empowerment would be computed by evolution or during the adaptation of the concrete organism. In an artificial computational model one can carry out explicit calculations of the quantities associated with empowerment. However, in biology a phenomenon such as empowerment maximization may originally play a guiding role in the discovery of novel sensorimotor modalities, but may then, on the long run, be effectively condensed over time into the form of natural homeostatic drives such as hunger, pain avoidance, or temperature regulation. In particular, the consideration of a universal utility such as empowerment does not necessarily provide us with a biological mechanism, but only with a principle. On the other hand, attaining such a principle could be enormously beneficial as it could be useful to make predictions, to guide our search for the concrete underlying biological mechanisms or even to help construct plausible biologically inspired artificial systems.

**Table 1:** Parameters to the dynamics of the pole-cart system.

Parameter	Value
Gravitational acceleration (*g*)	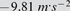
Length of half of the pole (*l*)	0.5 *m*
Mass of the cart ( 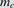 )	1 *kg*
Mass of the pole ( 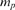 )	0.1 *kg*
Control force (*F*)	10 *N*
Integration time step (δ)	0.02 *s*

Information regarding Figures 8 and 9 can be found in the Appendix S2 and S4.

## Supporting Information

Appendix S1 Information Theory
(0.10 MB DOC)

Appendix S2 Bayesian Model of the Perception-Action Loop
(0.05 MB DOC)

Appendix S3 Information Flow
(0.15 MB DOC)

Appendix S4 Interventional Conditionals and Contextual Empowerment
(0.15 MB DOC)
