# Supplementary material for: Keep Your Options Open: An Information-Based Driving Principle for Sensorimotor Systems
Source: PLoS One. 2008 Dec 24;3(12):e4018. doi: 10.1371/journal.pone.0004018 (PMC2607028; doi:10.1371/journal.pone.0004018)
Supplement: Appendix S1 — Information Theory (0.10 MB DOC) [file pone.0004018.s001.doc]

# Appendix S1

## Information Theory

### Introduction

In this section the central information-theoretic notions used in this paper are briefly introduced. For a detailed introduction to information theory [69] consult, for example, [70].

A *random variable* can assume various values with various probabilities. In this paper exclusively discrete random variables are considered; where continuous state spaces appear originally, they are suitably quantized before being used as random variables.

Denote random variables with uppercase letters, e.g.,$X$, their sets of values with upright letters, e.g.,, and their values with lowercase letters, e.g., *x*. Denote composite random variables by listing their elements inside parenthesis, e.g.,$(X,Y)$with values$(x,y)$ from the set . By abuse of notation we denote$\Pr(X=x)$, the probability that *X* assumes the value *x*, by$p(x)$. Similarly, the joint probability of *X* and *Y* is denoted by$p(x,y)$ and the conditional probability of *X* given *Y* by$p(x|y)$.

### Entropies, Mutual Information, Channel Capacity

The *entropy* of *X*, denoted by *H*(*X*), is defined as a measure of the uncertainty of the probability distribution of *X*:

Entropy as well all other information-theoretic measures used in this paper are measured in *bits*. Note that all of the information-theoretic measures presented in this section are *non-negative*.

The *conditional entropy* of *X* given *Y*, denoted $H(X|Y)$, is defined as uncertainty of *X* knowing *Y* weighted by the probability of *Y*:

The *mutual information* between *X* and *Y*, denoted$I(X;Y)$, is defined as the average reduction in the uncertainty of *X* given *Y*:

$$I(X;Y):=H(X)-H(X|Y) .$$

It is symmetric in *X* and *Y*.

*Channel capacity* of a communication channel: consider a channel $X\to Y$from a variable *X* to a variable *Y*, characterized by the fixed given conditional$p(y|x)$. If a distribution $p(x)$ on *X* is given, consider$I(X;Y)$, the mutual information between *X* and *Y*. The channel capacity of that channel is the maximum of this mutual information over all possible input distributions $p(x)$ on *X*, and limits the amount of information that can be reliably transmitted through the channel: .

Consult also App. S3 for a more complete formalism of the causal aspects of a channel.
