# Supplementary material for: Keep Your Options Open: An Information-Based Driving Principle for Sensorimotor Systems
Source: PLoS One. 2008 Dec 24;3(12):e4018. doi: 10.1371/journal.pone.0004018 (PMC2607028; doi:10.1371/journal.pone.0004018)
Supplement: Appendix S2 — Bayesian Model of the Perception-Action Loop (0.05 MB DOC) [file pone.0004018.s002.doc]

# Appendix S2

## Bayesian Model of the Perception-Action Loop

We model the perception-action loop of an agent as a *causal* Bayesian network [42]. A Bayesian network is used to model statistical relationships between observed variables in a compact graphical way. In general, more than one Bayesian network may fit observed data. In a *causal* Bayesian network, the relationships between variables are not just statistical but also causal, corresponding to the underlying mechanisms that generate the data. This allows one to calculate effects of interventions – defined external changes in selected mechanisms (consult [42] for an in-depth treatment). Examples of interventions include fixing a variable to a particular value, or “injecting” information into the system (see below). The latter is used extensively in this paper. In general, interventions can only be defined for *causal* Bayesian networks.

Using the causal Bayesian network formalism allows us to formulate a general and minimal model of an agent with a controller, possibly with memory. The following assumptions are made in our model:

• the agent is part of a larger agent-environment system;

• the system has discrete states;

• the system is discrete in time;

• consecutive states of the system form a Markov chain – the momentary state of the system makes the past of the system statistically independent from its future; and

• the agent’s controller selects actions and modifies its own memory having access only to the momentary state of the sensors and its own memory.

There are many ways to partition such a system into subsystems. Here the partitioning is done from the perspective of the agent’s controller. The controller has direct access only to the agent’s sensor, actuator, and its own memory. Everything else in the system that is not encompassed by these three components is the rest of the system.

Note that, although the agent can have more than one sensor and actuator, from now on they will be referred to in singular. Multiple sensors are treated here as one composite sensor, but the formalism is not limited to that. Similar reasoning applies to actuators.

All the constituents of the perception-action loop are modeled as random variables:

• *S* – the state of the sensor;

• *A* – the action performed by the actuator;

• *M* – the state of the controller’s memory;

• *R* – the state of the rest of the system.

*R* formally accounts for the effects of actuation, the agent’s environment, and morphology on the sensors.

Sensors and actuators enable information flows between the agent and its environment. Time is introduced to model the temporal aspect of the flows. The states of the sensor, the actuator, the controller’s memory, and the rest of the system at discrete time *t* are denoted by random variables$S_{t}$, $A_{t}$, $M_{t}$, and $R_{t}$ respectively.

Modeling relations between the variables at different time steps *unrolls* the perception-action loop in time. The idea to treat an agent’s control loop information-theoretically dates back to Ashby [1], but has been little used otherwise until revived by Touchette and Lloyd [2, 71]. For our model, we use the formalism from [9] which significantly generalizes the earlier approaches to arbitrary time sequences or processing structures.

In this model, the relationships between the variables are modeled as a *causal Bayesian network* [42] which is a directed acyclic graph where any node, given its parents, is conditionally independent from any other node which is not its successor (any node directly or indirectly reachable from the node).

The basic causal Bayesian network used to model an agent represents a pattern of relations between variables at consecutive time steps and is shown in Fig. 8. We assume that the pattern of relations is time-invariant and thus holds for any *t*. Thus, it is sufficient to consider a graph as in Fig. 8 which represents just a section of the network.
