# Supplementary material for: Keep Your Options Open: An Information-Based Driving Principle for Sensorimotor Systems
Source: PLoS One. 2008 Dec 24;3(12):e4018. doi: 10.1371/journal.pone.0004018 (PMC2607028; doi:10.1371/journal.pone.0004018)
Supplement: Appendix S3 — Information Flow (0.15 MB DOC) [file pone.0004018.s003.doc]

# Appendix S3

## Information Flow

### Definition

Given a causal Bayesian network, define information flow from a random variable *X* to a random variable *Y*, denoted by$I(X\to Y)$, as the amount of information about *X* causally transmitted from *X* to *Y* [41]. Without loss of generality *X* and *Y* can be compound variables consisting of multiple random variables.

To do this, we need Pearl’s interventional calculus and his definition of *causal effect* of *X* on *Y*, denoted by$p(y|\hat{x})$which, for every realization of *X*, describes the probability distribution of *Y* if *X* is set to that realization *x*, by modifying the Bayesian network through detaching *X* from its parents and forcing its value to be *x* (see [42] for more details). For any but the simplest Bayesian network$X\to Y$, we need to consider$p(y|\hat{x})$as the description of the *communication channel* from *X* to *Y*, with *X* the *input*, and *Y* the *output* of the channel. If *X* is instantiated with a probability distribution$p(x)$, then the average amount of information about *X* transferred over the channel to *Y* is simply$I(X;Y)$, the mutual information between *X* and *Y*. The mutual information can then be obtained from$p(x)$and$p(y|\hat{x})$.

The above quantification of amount of information transferred over the channel assumes that *X* is set to a particular value distribution independently of the rest of the system’s state. We want to define information flow also for cases when *X* is observed rather than set. In this case it may no longer be possible to “plug in”$p(x)$ into$p(y|\hat{x})$to obtain the amount of information flow.

The complication arises from the fact that the channel from *X* to *Y* may have state, i.e. there exists a non-empty set *Z* of non-descendants of *X*, such that $p(y|\hat{x},z)\neq p(y|\hat{x})$. $p(y|\hat{x})$ is obtained by assuming that *X* will be set independently of non-descendants of *X*, and thus independently of the state of the channel. In the general case, when *X* may depend on its non-descendants, *X* may therefore be correlated with the state of the channel, and hence one must be careful when using $p(y|\hat{x})$ to quantify the information flow.

Define here information flow for the simplest case when *X* is not correlated with the state of the channel – an approach to the more general treatment can be found in [41]. If *X* is *d*-separated from *Y* in$G_{\underline{X}}$, i.e. in the Bayesian network resulting from the removal of all outgoing arrows from *X*, then the information flow from *X* to *Y* is given as:

$I(X\to Y)=I(X;Y)|_{p(y|\hat{x})} ,$ (13)

where$I(X;Y)|_{p(y|\hat{x}}$ is the mutual information obtained from $p(y|\hat{x})$ as channel characteristics and *p*(*x*) as input distribution. In this special case$p(y|\hat{x})=p(y|x)$, hence$I(X;Y)|_{p(y|\hat{x})}=I(X;Y)$. Examples of matching networks include *X* being an exogenous variable, or *X* and *Y* being nodes in a data-processing chain. Importantly, note that the calculation of empowerment as maximum potential information flow does not suffer from this restriction as it requires the source variable *X* to be driven by an independent external source of information *Z*.

### Contextual Information Flow

Given a context $C$ which is a compound random variable consisting of arbitrary non-descendants of $X$, define *contextual information flow* from $X$ to $Y$ given the context $C$, denoted by $I(X\to Y|C)$, as the average information flow from $X$ to $Y$ when $C=c$ weighted by the probability of $C=c$ (see again [41], and also App. S4 for related quantities):

(14)

The contextual information flow obviously depends on the choice of context. The context-free information flow defined in the previous section is a special case where the context is empty.

Applying a channel state argument similar to the one used in the previous section for the quantification of information flow, if $X$ is $d$-separated from $Y$ by $C$ in $G_{\underline{X}}$, then

$I(X\to Y|C)=I(X;Y|C) .$ (15)

Again, empowerment, with its externally driven input, is not affected by this constraint.
