# Supplementary material for: Keep Your Options Open: An Information-Based Driving Principle for Sensorimotor Systems
Source: PLoS One. 2008 Dec 24;3(12):e4018. doi: 10.1371/journal.pone.0004018 (PMC2607028; doi:10.1371/journal.pone.0004018)
Supplement: Appendix S4 — Interventional Conditionals and Contextual Empowerment (0.15 MB DOC) [file pone.0004018.s004.doc]

# Appendix S4

## Interventional Conditionals and Contextual Empowerment

Here we detail the formulation of the context-dependent empowerment calculation. For this purpose, we introduce some notation, first for the context-less empowerment.

Consider Fig. 9. Then the empowerment is calculated as

$$\equiv\max_{p(a_{t},\ldots,a_{t+n-1})} I_{\bar{p}}(A_{t}^{n};S_{t+n}) ,$$

where $(A_{t},\ldots,A_{t+n-1},S_{t+n})\equiv(A_{t}^{n},S_{t+n})$ are distributed according to

$$\bar{p}(a_{t},\ldots a_{t+n-1},s_{t+n})=p(s_{t+n}|\hat{a}_{t},\ldots\hat{a}_{t+n-1}) p(a_{t},\ldots a_{t+n-1}) ,$$

with $p(s_{t+n}|\hat{a}_{t},\ldots\hat{a}_{t+n-1})$ being the usual interventional conditional distribution according to the given Bayesian network, in our case that from Fig. 9. Note that here we use the notation $I_{p}(X;Y)$ for the mutual information to make the joint distribution $p(x,y)$ linking two random variables $X$ and $Y$ explicit.

We now extend this formulation to context-dependent empowerment. A *context*, denoted with a random variable $C$, is a collection of random variables from the causal Bayesian network which are non-descendants of the variables in $A_{t}^{n}$ (and $Z_{t}$). The empowerment , given a particular fixed observation $c$ of $C$, is then calculcated by

where $(A_{t}^{n},S_{t+n})$ are distributed according to

$$\bar{p}_{c}(a_{t},\ldots a_{t+n-1},s_{t+n})=p(s_{t+n}|\hat{a}_{t},\ldots\hat{a}_{t+n-1},c) p(a_{t},\ldots a_{t+n-1}) ,$$

with

$$p(s_{t+n}|\hat{a}_{t},\ldots\hat{a}_{t+n-1},c)=\frac{p(s_{t+n},c|\hat{a}_{t},\ldots\hat{a}_{t+n-1})}{p(c|\hat{a}_{t},\ldots\hat{a}_{t+n-1})}$$

and, since $\text{C}$ is non-descendant of $\text{A}_{\text{t}}^{\text{n}}$, it is causally independent from the latter and thus

$$=p(s_{t+n},c|\hat{a}_{t},\ldots\hat{a}_{t+n-1})/p(c) .$$

In the case that also $S_{t+n}$ is non-descendant of $A_{t}^{n}$, then $(S_{t+n},C)$ is causally independent from $A_{t}^{n}$ and $p(s_{t+n}|\hat{a}_{t},\ldots\hat{a}_{t+n-1},c)=p(s_{t+n},c)/p(c)=p(s_{t+n}|c)$, and thus the contextual empowerment vanishes. This shows in particular that a context does not introduce spurious non-causal dependencies. Finally, the total contextual empowerment is attained as an probabilistically weighted average over the different realized contexts.

(16)

In the pole-balancing as well as the sensor evolution, we use a special context, namely the full current world state of the system, before the actions are selected (represented by $R_{t}$ in Fig. 9). Since the agent itself has no memory in these scenarios, the full world state $R_{t}$ forms a maximal context.

In these scenarios, we determined the interventional dynamics of the actions in various world states (i.e. contexts) by starting the system in these states and then determining the subsequent effect of the agent's actions. Note that this procedure is legitimate, as the interventional distribution of the actions attained in this case is equivalent to that obtained by a passive observation of states, i.e.

$p(s_{t+n}|\hat{a}_{t},\ldots\hat{a}_{t+n-1},\hat{c})=p(s_{t+n}|\hat{a}_{t},\ldots\hat{a}_{t+n-1},c)$ (17)

(as long as in the latter case the contexts - i.e. states - are visited with nonvanishing probability). This follows from Rule 2 of Pearl's interventional calculus [42, Theorem 3.4.1, page 85]; in our situation, this rule essentially states that if there exists no ``backdoor'' between the current world state $R_{t}$ and the future sensoric states that circumvents the intervening actions, an interventional context is equivalent to an observational one. In our scenarios this property holds true as we assume the world state to be a complete description of the environment.

For the concrete computation of the interventional distribution in the simulations, one can place the agent in various states in the world and operate randomized actions, whereby one will obtain the interventional distribution $p(s_{t+n}|\hat{a}_{t},\ldots\hat{a}_{t+n-1},\hat{c})$ from measuring the empirically obtained relative frequencies.

If this is not possible or desirable, but one has access to full information for each time slice (as is typically the case in simulations), then, for a single interventional node, the interventional distributions can also be computed from purely observational data by controlling for the parents of the interventional node [42, 41]. Denote by $\text{Pa}(\mathbf{A}_{t})$ the *parents* of the node $\mathbf{A}_{t}$, i.e. the set of immediate predecessors of the node $\mathbf{A}_{t}$ in the Bayesian graph and by $\text{Pa}(\mathbf{a}_{t})$the values they assume. Then, one has the following term for the interventional conditional of the sensoric values for given actions:

$$p(s_{t+n}|\hat{\mathbf{a}}_{t})=\sum_{\text{Pa}(\mathbf{a}_{t})} p(s_{t+n}|\mathbf{a}_{t},\text{Pa}(\mathbf{a}_{t})) p(\text{Pa}(\mathbf{a}_{t}))$$

where we have dropped the context-dependence for the sake of simplicity. Note that actually not even the knowledge of the full time slice $t-1$ is necessary to compute this interventional quantity, only the parents of the action. However, one thing that is necessary to ensure is that the action attains all possible interventional values. Whenever full time slices are not available or controlling for the parents is not possible, one has to resort to approximations, based on suitable assumptions; an example for such an approach is shown in the AIBO experiment. If one has more than one action node, then the computation of the interventional distribution only from observations is in general more involved, if at all possible. In this case, one has in general to resort to applying the full-fledged interventional calculus from [42].
